# Differential Expression of *Yersinia pseudotuberculosis* General Porin Genes during Short- and Long-Term Antibiotic Stresses

**DOI:** 10.3390/molecules26133956

**Published:** 2021-06-28

**Authors:** Evgeniya Bystritskaya, Nadezhda Chernysheva, Anna Stenkova, Konstantin Guzev, Alexander Rakin, Marina Isaeva

**Affiliations:** 1 G.B. Elyakov Pacific Institute of Bioorganic Chemistry, Far Eastern Branch, Russian Academy of Sciences, 159, Pr. 100 Let Vladivostoku, 690022 Vladivostok, Russia; belyjane@gmail.com (E.B.); chernysheva.nadezhda@gmail.com (N.C.); k.guzev@gmail.com (K.G.); 2 School of Biomedicine, Far Eastern Federal University, 8 Sukhanova St., 690090 Vladivostok, Russia; stenkova@gmail.com; 3 Friedrich-Loeffler-Institut, Federal Research Institute for Animal Health, Institute for Bacterial Infections and Zoonoses, Naumburger Str. 96a, D-07743 Jena, Germany; rakin@gmx.de

**Keywords:** porin gene expression, *Yersinia pseudotuberculosis*, antibiotic stress, phenotypic heterogeneity

## Abstract

Here, we investigated general porin regulation in *Yersinia pseudotuberculosis* 488, the causative agent of Far Eastern scarlet-like fever, in response to sublethal concentrations of antibiotics. We chose four antibiotics of different classes and measured gene expression using qRT-PCR and GFP reporter systems. Our data showed temporal regulation of the general porin genes *ompF* and *ompC* caused by antibiotic stress. The porin transcription initially decreased, providing early defensive response of the bacterium, while it returned to that of the untreated cells on prolonged antibiotic exposure. Unlike the major porin genes, the transcription of the alternative porin genes *ompX* and *lamB* was increased. Moreover, a short-term *ompR*- and *marA*-mediated porin regulation was observed. The main finding was a phenotypic heterogeneity of *Y. pseudotuberculosis* population manifested in variable porin gene expression under carbenicillin exposure. This may offer adaptive fitness advantages for a particular bacterial subpopulation.

## 1. Introduction

The global rise of antibiotic resistance in pathogens represents a serious threat to medicine, as it is the predominant cause of treatment failure and increased mortality [1]. Recent evidence suggests that sublethal concentrations of antimicrobials frequently found in the human body and nature play an important role in the development of antibiotic resistance [2]. Initially, susceptible bacterial populations survive and propagate under such conditions due to adaptive gene expression responses that have been observed in a variety of bacterial species [1,3,4,5]. In Gram-negative bacteria, altering the gene expression of outer membrane proteins or porins, resulting in decreased membrane permeability, is considered one of the first defense mechanisms of adaptation to antibiotic stress [1].

*Yersinia pseudotuberculosis*, a Gram-negative bacterium belonging to the *Enterobacteriaceae* family, is a human pathogen causing pseudotuberculosis infection. In Russia and Japan, it causes outbreaks of the disease known as Far Eastern scarlet-like fever (FESLF), with serious systemic inflammatory symptoms [6].

*Y. pseudotuberculosis* produces two major nonspecific porins, OmpF and OmpC, which we study intensively [7,8,9,10,11]. These porins consist of 16-stranded β-barrel trimers, each of which forms a central channel [12]. The channels control the permeability of the cell envelope for low molecular compounds, including β-lactam, tetracycline, chloramphenicol, and fluoroquinolone antibiotics [13,14,15,16]. Thus, the porins provide cell defense against certain antibiotics and subsequently mediate antimicrobial resistance by downregulating their gene expression or inducing beneficial mutations [17]. Moreover, the reversible phenotypical response offers advantages over conventional irreversible mutations [18]. Furthermore, transcriptional porin regulation is considered a rapid adaptive mechanism to environmental and antibiotic stresses occurring within the first 60 min [19]. The porin-mediated stress response has been widely studied in various enterobacteria, including *Escherichia coli*, *Klebsiella pneumonia*, *Salmonella enterica*, *Serratia marcescens*, and others [20,21,22]. Still, little is known about the effect of antibiotic stress on the general porin regulation in *Y. pseudotuberculosis*. Most research focuses on the effect of lethal antibiotic concentrations on bacterial physiology and resistance, while subinhibitory antimicrobial doses have been mostly disregarded [23]. *Y. pseudotuberculosis* genetic response to low concentrations of antibiotics is of particular interest because successful treatment of pseudotuberculosis infection largely depends on antibiotic therapy to maintain lethal drug concentrations in different human tissues.

Therefore, we aim to investigate the role of general porin regulation in *Y. pseudotuberculosis* adaptation to sublethal concentrations of four antibiotics utilizing porin channels for cell entrance.

## 2. Results and Discussion

### 2.1. Antibiotics and Y. pseudotuberculosis 488 Susceptibility

*Y. pseudotuberculosis* 488 (= strain 117), serotype O:1b, was isolated from a patient with FESLF in the Russian Far East region. To elucidate *Y. pseudotuberculosis* porin regulation under antibiotic stress, we chose carbenicillin, tetracycline, kanamycin, and chloramphenicol. These antibiotics have been shown to use porin channels to enter into a bacterial cell [13,14]. Several studies have demonstrated that porin regulation, resulting in decreased penetration of antimicrobials into the cell, contributes to bacterial adaptive resistance [24]. We determined the MIC ranges of the selected antibiotics for *Y. pseudotuberculosis* 488 at 27 °C (optimal for bacterial cultivation) and 37 °C (human body temperature) since it is known that porin expression is tightly regulated by environmental factors [25,26], predominantly by temperature [27,28]. Such regulation is shown for *Y. pseudotuberculosis* strains in the interactive RNA atlas www.pathogenex.org (*ompF* YPK_2649; *ompC* YPK_2839).

*Y. pseudotuberculosis* 488 showed high susceptibility to the tested antibiotics (Table S1). However, cells cultivated at 27 °C exhibited a 2-fold increased resistance to kanamycin and tetracycline compared with 37 °C, whereas the MICs of carbenicillin and chloramphenicol were not affected by incubation temperature. To induce antibiotic stress, we used subinhibitory concentrations (sub-MICs) of antibiotics corresponding to ½ MICs. Such sublethal antibiotic concentrations reflect the conditions that bacteria may encounter in the natural environments and the human body [29]. Moreover, exposure to sublethal concentration has been reported to play a protective role in the bacterial cell against a wide range of antimicrobials and alter the expression of various bacterial genes that can lead to nonspecific resistance [22].

### 2.2. An Early Transcriptional Response of General Porin Genes to Antibiotic Stress

Rapid modulation of porin gene expression is the first line of bacterial defense against harmful compounds, including antibiotics, which increases the chances of survival and adaptation of microorganisms to stressful conditions [19].

**Figure 1:**
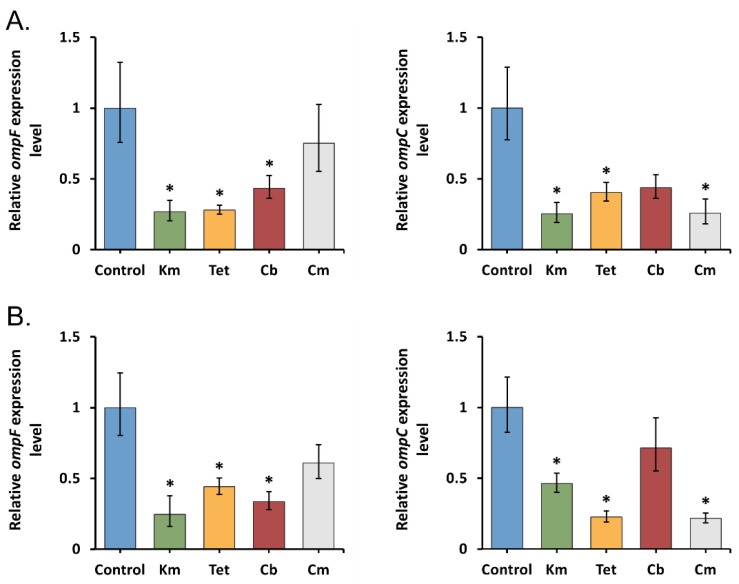
Relative expression levels of *Y. pseudotuberculosis ompF* and *ompC* after short-term antibiotic exposure detected by qRT-PCR. (**A**) 27 °C incubation temperature; (**B**) 37 °C incubation temperature. All results are expressed as mean ± standard deviation from three independent experiments. An asterisk indicates *p*-value < 0.05 vs. respective untreated control. Significance was calculated using one-way ANOVA. Km—kanamycin, Tet—tetracycline, Cb—carbenicillin, and Cm—chloramphenicol.

To study the early porin response in *Y. pseudotuberculosis* 488, we treated bacterial cells by sub-MICs of antibiotics for an hour, followed by measuring the level of *ompF* and *ompC* transcripts using qRT-PCR. The results obtained (Figure 1) indicate that kanamycin and tetracycline cause a transcription decrease in both general porins, regardless of temperature. *ompF* and *ompC* expression were downregulated 2.3- to 4-fold and 2.2- to 4.4-fold, respectively, in the presence of these antibiotics. It is not surprising because the reduced porin expression is considered to be a common strategy for protection against antibiotics. However, tetracycline and kanamycin downregulated only OmpF synthesis and upregulated OmpC production in *Escherichia coli* [30,31,32,33]. Nevertheless, Agafitei et al. have shown that reduced expression of the OmpC porin contributes to kanamycin resistance [34]. Viveiros et al. also found a coupled downregulation of the *E. coli* OmpF with the OmpC during exposure to increasing concentrations of tetracycline [24].

Interestingly, two other antibiotics caused different transcriptional responses of the porins in *Y. pseudotuberculosis* 488. After one-hour exposure of cells to carbenicillin, the expression level of *ompF* showed a 2.3- to 3-fold decrease, while the expression of *ompC* was not changed significantly (Figure 1). This result supports the findings that OmpF is the preferred route of entry for β-lactams [35]. We suppose that a decrease of *ompF* mRNA level observed at both temperatures could be a protective response of *Y. pseudotuberculosis* cells to the carbenicillin stress. This opinion agrees with the previous finding that lack or inhibition of OmpF contributes to increased resistance to β-lactams [36,37]. It was also unexpected that under short-term chloramphenicol stress, the *ompF* expression was not significantly altered, whereas the *ompC* expression was downregulated 3.9 to 4.6 times (Figure 1). Chloramphenicol has been reported, unlike β-lactams, to utilize both porins equally well [16]. However, there is no observation to indicate any effect of a reduced level of OmpC alone on the penetration of this antibiotic [38].

Considering the qRT-PCR results, we assumed that the general porins OmpF and OmpC of *Y. pseudotuberculosis* are involved in the early response to various antibiotic stresses caused by sublethal concentrations and provide the cells with the first defense mechanism by reducing the transcription level of one or both of their genes depending on the antibiotic type.

### 2.3. Transcriptional Response of General Porin Genes to Prolonged Antibiotic Stress

Several studies consider the modulation of porin gene expression as a rapid but temporary response of the cell to stress, which develops within the first 60 min [19,23]. However, in the case of antibiotic treatment, this mechanism can be replaced by more specific ones over time [1]. The data on the porin gene expression in time series are important for a comprehensive understanding of *Y. pseudotuberculosis* defense against different antimicrobials.

**Figure 2:**
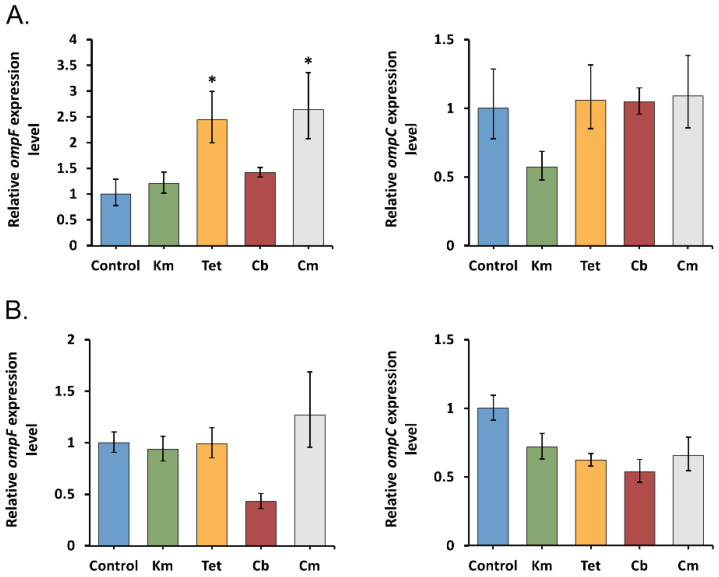
Relative expression levels of *Y. pseudotuberculosis ompF* and *ompC* after prolonged antibiotic exposure detected by qRT-PCR. (**A**) 27 °C incubation temperature; (**B**) 37 °C incubation temperature. All results are expressed as mean ± standard deviation from three independent experiments. An asterisk indicates *p*-value < 0.05 vs. respective untreated control. Significance was calculated using one-way ANOVA. Km—kanamycin, Tet—tetracycline, Cb—carbenicillin, and Cm—chloramphenicol.

To evaluate the effect of prolonged antibiotic exposure on the porin gene expression, *Y. pseudotuberculosis* 488 was cultivated in the presence of sub-MICs of these antibiotics for 16 h. In contrast to the early porin response, here we did not see significant changes in the expression of *ompF* and *ompC* genes in most samples treated with antibiotics (Figure 2). The data obtained indicate that transcription of general porins in *Y. pseudotuberculosis* cells transiently decreased within the first hour and subsequently returned to levels comparable to those of the untreated cells.

Previously, Viveiros et al. observed that *ompF* and *ompC* transcript levels in *E. coli* remained comparable to the untreated controls after prolonged exposure to tetracycline [24]. However, post-translational *marA*-mediated porin regulation to antibiotic stress has been shown to inhibit their synthesis.

Surprisingly, we found a 2.4- and 2.6-fold increase in the expression of *ompF* under prolonged tetracycline and chloramphenicol stresses at 27 °C, which no one had reported earlier. At the same time, incubating cells with these two antibiotics at 37 °C did not affect porin transcription. One of the possible reasons for the *ompF* upregulation in the presence of tetracycline and chloramphenicol could be the decreased expression of alternative porins.

We hypothesized that observed changes in the porin regulation under antibiotic stress over time could be caused by several factors. For example, stationary-phase bacteria must respond and adapt to a variety of environmental stresses (nutrient exhaustion, pH changes, oxygen limitation, and others). In such a case, the regulation of porins provides the balance of outer membrane permeability between self-defense and competitiveness [39,40]. The significantly reduced expression of general porins, protecting cells from antibiotics within the first hour, might entail high metabolic costs at the stationary phase, since vital nutrients are simultaneously excluded.

It should be noted that in addition to the transcriptional regulation of porin genes, post-transcriptional regulation might play a major role in the physiological adaptation to prolonged antibiotic exposure, as was mentioned previously [24].

Moreover, the acquisition of mutations in porin genes, leading to loss of proteins, size, and conductance modification of their channels or decreasing their expression level, is known as another common mechanism for reducing the sensitivity of bacteria to antibiotics. It was observed that porin-related mutations substantially influence resistance to β-lactams, fluoroquinolones, tetracycline, and chloramphenicol [1]. However, we performed *ompF* and *ompC* sequence analysis of *Y. pseudotuberculosis* samples after prolonged antibiotic treatment and did not reveal any changes within the porin-encoding parts and their regulatory regions.

### 2.4. ompR and marA Effect on Porin Response under Antibiotic Stress

Next, we investigated the contribution of transcriptional factors in the regulation of porin genes.

The best-described input into controlling porin levels involves OmpR, which regulates *ompC* and *ompF* directly through OmpR–promoter DNA association [41]. The OmpR/EnvZ two-component system may play a major role in antibiotic resistance by modulating porin expression [42]. It was shown that OmpR deficiency in *Y. pseudotuberculosis* and *Y. enterocolitica* reduces bacterial sensitivity to the β-lactams, tetracycline, and polymyxin B, caused by the lack of OmpF and OmpC porins [43,44].

**Figure 3:**
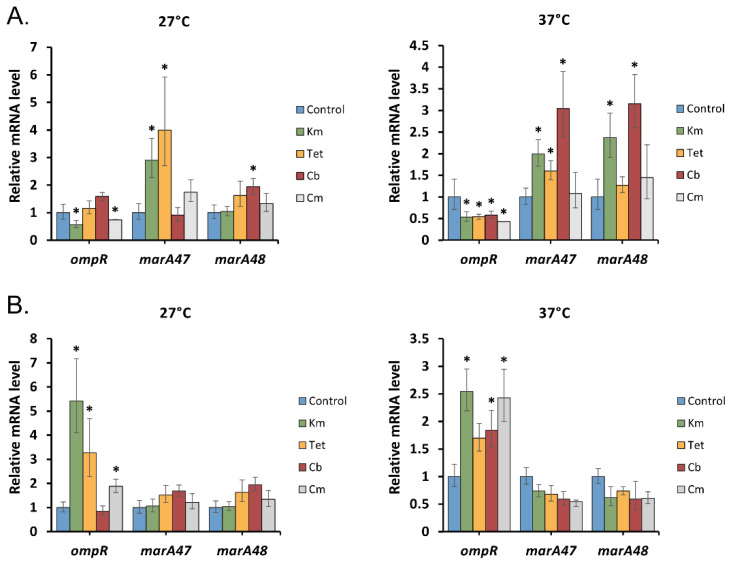
Relative expression levels of *Y. pseudotuberculosis ompR*, *marA47*, and *marA48* under antibiotic stress detected by qRT-PCR. (**A**) 1 h antibiotic exposure, (**B**) 16 h antibiotic exposure. All results are expressed as mean ± standard deviation from three independent experiments. An asterisk indicates *p*-value < 0.05 vs. respective untreated control. Significance was calculated using one-way ANOVA. Km—kanamycin, Tet—tetracycline, Cb—carbenicillin, and Cm—chloramphenicol.

qRT-PCR analysis revealed differential expression of *ompR* in *Y. pseudotuberculosis* depending on the duration of antibiotic stress (Figure 3). Short exposure to antibiotics excluding carbenicillin and tetracycline at 27 °C slightly reduced (1.7- to 2.3-fold) its transcription associated with the downregulation of porin genes. On the contrary, prolonged antibiotic stress increased the level of *ompR* transcripts up to 5.3-fold; however, this did not affect the expression of *ompF* and *ompC*. A similar observation was described in the work of Viveiros et al., where the response of the *ompC* and *ompF* genes, despite the increased expression of *ompR*, remained comparable to their levels in *E. coli* cells not exposed to tetracycline [24]. Moreover, it was shown that OmpR positively controls the expression of the AcrAB-TolC efflux pump involved in the adaptive response of the *Y. enterocolitica* strain to different chemical stressors [45]. It is tempting to speculate that a high level of *ompR* transcription in *Y. pseudotuberculosis* may be required to induce this efflux pump system as another general defense mechanism against antibiotics.

Multiple studies have shown that general porin expression is post-transcriptionally regulated by global regulator MarA, involved in multidrug resistance response during antibiotic stress [46,47]. *marA* locus can directly or indirectly increase the level of *micF*, a non-coding RNA stress response gene, causing a post-transcriptional downregulation of *ompF* mRNA and reducing the OmpF synthesis [47]. Two MarA-like proteins have been reported in *Yersinia pestis*: MarA47 and MarA48 [48,49].

To test their involvement in the regulation of *Y. pseudotuberculosis* porin expression under short- and long-term exposure to antibiotics, we measured the levels of *marA47* and *marA48* mRNA using qRT-PCR (Figure 3). We found different activities of *marA* transcripts depending on the time of antibiotic exposure. A short treatment of cells with the antibiotics caused upregulation of at least one *marA* gene, except for chloramphenicol, which did not affect the *marA* expression. In the case of long-term exposure to the tested antibiotics, the level of *marA* expression remained comparable to those of the control samples.

The obtained results led us to conclude that regulation of *Y. pseudotuberculosis* general porins mediated by MarA was observed only for early antibiotic stress caused by sublethal concentrations of tetracycline, kanamycin, and carbenicillin. During long-term antibiotic presence, *marA* transcription remained unaltered and therefore MarA did not play a significant role in porin regulation at the post-transcriptional level.

### 2.5. Transcriptional Response of Y. pseudotuberculosis Alternative Porins to Prolonged Antibiotic Stress

In addition to major porins, *Enterobacteriaceae* can express alternative outer membrane proteins that may contribute to the adaptation of bacteria to antibiotic stress by modulating OmpF and OmpC levels or directly changing the cell permeability [35,50].

Therefore, to better understand the mechanism of *Y. pseudotuberculosis* adaptation, we investigated the transcriptional response of the genes coding for OmpX, OmpA, LamB, and OmpY porins to prolonged antibiotic stress.

**Figure 4:**
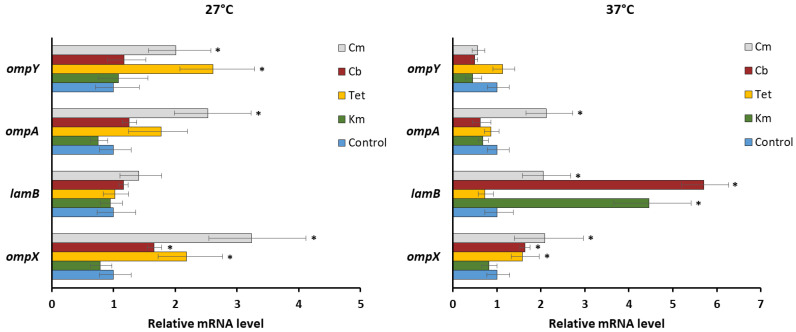
Relative expression levels of *Y. pseudotuberculosis ompX*, *lamB*, *ompA*, and *ompY* under prolonged antibiotic stress detected by qRT-PCR. All results are expressed as mean ± standard deviation from three independent experiments. An asterisk indicates *p*-value < 0.05 vs. respective untreated control. Significance was calculated using one-way ANOVA. Km—kanamycin, Tet—tetracycline, Cb—carbenicillin, and Cm—chloramphenicol.

Using qRT-PCR, we observed the increased activity of *ompX* transcripts (1.7–3.2-fold) in the presence of most antibiotics (Figure 4), which is consistent with previously reported results [50]. It is believed that overexpression of OmpX may impair the normal assembly of major porins, which leads to their subsequent degradation by Deg proteases [51]. From this, we hypothesized that OmpF and OmpC expression of *Y. pseudotuberculosis* could be regulated by this mechanism at the post-translational level.

We also observed the induction of *lamB* transcription in the presence of kanamycin, carbenicillin, and chloramphenicol at 37 °C (Figure 4), when its initial mRNA level was significantly lower compared to 27 °C (data not shown). It is interesting to note that overexpression of LamB previously found for *Enterobacter aerogenes*-resistant isolates was associated with a low amount of its major porins [52].

The transcription of *ompA*, playing a more important role in the maintenance of membrane integrity, rather than antibiotic transport [35], remained unaltered in *Y. pseudotuberculosis*, except for chloramphenicol exposure, where the *ompA* expression increased up to 2.5-fold (Figure 4).

Previously, we described a new quiescent porin OmpY in *Y. pseudotuberculosis* initially predicted from the genomic data [53]. To date, our knowledge of such porins has been very limited. Both the functional role of OmpY and the conditions significantly affecting its expression in *Yersinia* are still unknown. qRT-PCR analysis revealed *ompY* upregulation under chloramphenicol and tetracycline exposure at 27 °C, while at 37 °C its level did not change (Figure 4). Several studies have shown that the expression of quiescent porins was induced by antibiotics; however, the impact of such regulation on antibiotic susceptibility remains questionable [20,54].

### 2.6. ompF and ompC Expression within Y. pseudotuberculosis Population

Current experimental evidence suggests that bacterial cultures are constituted of heterogeneous subpopulations [55] with differential responses to environmental stresses [56,57]. For instance, Sánchez-Romero and Casadesús have observed the population heterogeneity of *S. enterica* cells in the expression level of *ompC* under kanamycin exposure [57]. Moreover, a low level of *ompC* correlates with high kanamycin resistance of the cell.

Our qRT-PCR results based on average C_t_ values did not reveal any significant transcriptional response of porins to long-term antibiotic exposure for most samples. They said nothing about the variability of *ompF* and *ompC* expression in the *Y. pseudotuberculosis* population or the amount of their gene products in the cell. To answer these questions, we first developed fluorescent reporter strains of *Y. pseudotuberculosis* 488. The GFP reporter systems were constructed by fusion of the *ompF*/*ompC* regulatory regions, including binding sites for transcription and translation factors (5′UTRs and signal sequences for sRNAs, C-terminal YQF amino acids for proteases), to the GFP reporter gene in a low copy number plasmid pACYC184 (Figure S1). Thus, the fluorescence of *Y. pseudotuberculosis* transformants potentially not only provided a high-resolution method for assaying *ompF*/*ompC* promoter activity but also indirectly indicated OmpF and OmpC amounts. We excluded chloramphenicol from the experiments due to the plasmid containing the gene for its resistance. However, we additionally verified that the presence of this antibiotic in the medium did not affect the level of sample fluorescence (Figure S2).

**Figure 5:**
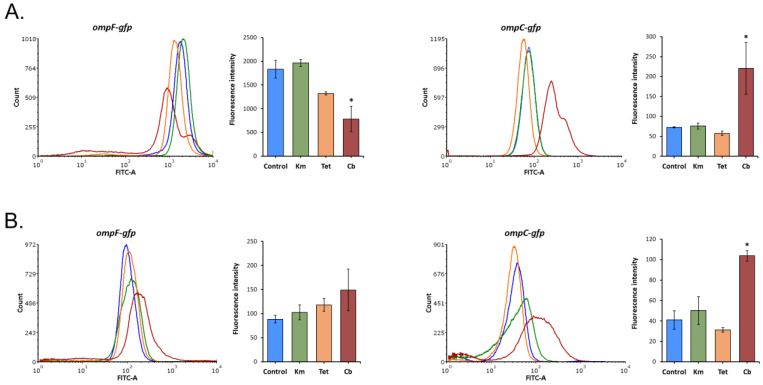
GFP fluorescence intensity in *Y. pseudotuberculosis* 488 transformed with *ompF*/*ompC* promoter-fused GFP reporter constructs following 16 h antibiotic treatment monitored by flow cytometry. (**A**) 27 °C incubation temperature; (**B**) 37 °C incubation temperature. All results are expressed as mean ± standard deviation between three experimental trials. An asterisk indicates *p*-value < 0.05 vs. respective control. Significance was calculated using one-way ANOVA. The histograms from a single representative experiment are shown. Control—without antibiotic treatment, Km—kanamycin, Tet—tetracycline, and Cb—carbenicillin.

There was little evidence of any change in the mean fluorescence intensity, and hence OmpF and OmpC expression, after prolonged exposure to tetracycline and kanamycin (Figure 5), while qRT-PCR revealed the *ompF* upregulation in the presence of tetracycline at 27 °C (Figure 2). This difference indicates an additional post-transcriptional regulation of OmpF.

After treatment with sub-MIC of carbenicillin at 27 °C, there was a 2.3-fold decrease in the *ompF* level and, on the contrary, a 2.5–3-fold increase in the *ompC* level. This is consistent with the previous observation that adaptation to β-lactams can involve increased expression of OmpC relative to OmpF since an increase in OmpC proportion hinders their penetration [37]. It is worth noting that carbenicillin inhibited the expression of *ompF* only at 27 °C. We observed that this transcript was prevailing compared to *ompC* in the untreated samples at both incubation temperatures; however, at 37 °C, its level decreased by more than 18 times (Figure S3). This initially low amount of *ompF* transcripts and additional nutrient deficiencies due to the stationary growth phase could prevent a further decrease in its expression at 37 °C under carbenicillin stress.

**Figure 6:**
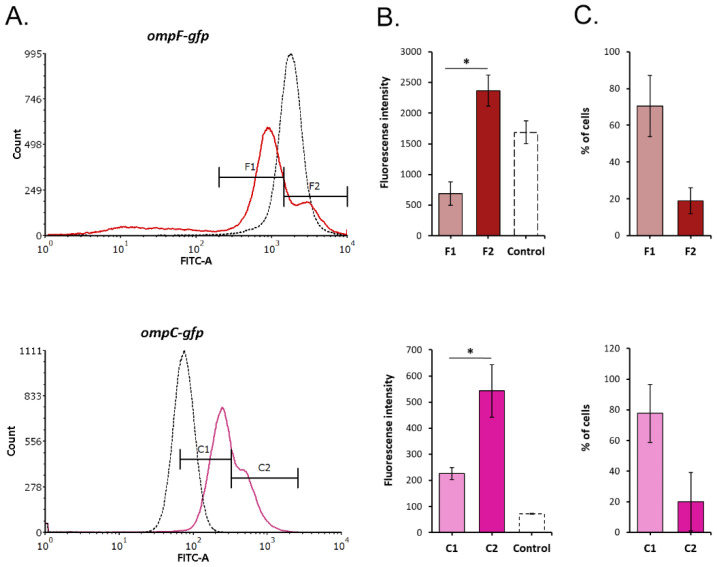
Fluorescence heterogeneity of *Y. pseudotuberculosis* 488 transformed with *ompF/ompC* promoter-fused GFP reporter constructs following treatment with a sublethal dose of carbenicillin monitored by flow cytometry. (**A**) Fluorescence histograms; (**B**) fluorescent intensity of subpopulations; (**C**) the percentage of cells in each subpopulation. All results are expressed as mean ± standard deviation between three experimental trials. An asterisk indicates *p*-value < 0.05 between groups. Significance was calculated using one-way ANOVA. The histograms from a single representative experiment are shown. F1, F2, and C1, C2—subpopulations of *Y. pseudotuberculosis* 488 with low/high GFP fluorescence.

The distribution of GFP fluorescence within the sample cultures under kanamycin and tetracycline exposure was comparable to the untreated controls. Only carbenicillin presence led to the appearance of two *Y. pseudotuberculosis* subpopulations with different fluorescence intensities, most notably at 27 °C (Figure 6). F1 subpopulation responded to carbenicillin stress by 1.9-fold inhibition of *ompF* expression, while the cells from F2 increased it by 1.8 times. *ompC* was upregulated under carbenicillin exposure in both subpopulations; however, in C2, its level was significantly higher. Moreover, most of the cells formed subpopulations with a lower level of both *ompF* and *ompC* expression (71% and 78% of the total cell amount, respectively; Figure 6C).

We thus propose that modification of the bacterial envelope by reduced *ompF*/increased *ompC* expression confers adaptive fitness advantages to carbenicillin in a fraction of the bacterial population.

Previously it was shown that heterogenous responses improve the survival of bacterial cultures under the effect of stress factors [58]. Moreover, gene expression variability, including porin genes, is considered to underly adaptive resistance in phenotypically heterogeneous microbial populations [57,59].

## 3. Materials and Methods

### 3.1. Bacterial Strain and Growth Conditions

*Y. pseudotuberculosis* 488 strain (= 117 strain, O:1b serotype) isolated from a patient with FESLF in the Russian Far East region was used in the present study.

To study short-term porin response to antibiotic stress, bacterial cells were grown in LB broth overnight at 27 °C with shaking. The next day, overnight cultures were diluted at a ratio of 1:50 into fresh LB medium and incubated with agitation at 27 °C (optimal growth temperature) and 37 °C (host temperature) to mid-exponential growth phase (OD_600_ = 0.4–0.6). Then, the antibiotics kanamycin (Sigma-Aldrich, St. Louis, MA, USA), tetracycline (Sigma-Aldrich, St. Louis, MA, USA), chloramphenicol (Sigma-Aldrich, St. Louis, MA, USA), and carbenicillin (Sigma-Aldrich, St. Louis, MA, USA) were added to the media in sublethal concentrations (0.5 MIC). After a 1 h antibiotic treatment (180 rpm, 27 °C and 37 °C), the cells were mixed with RNAprotect Bacteria Reagent (Qiagen, Hilden, Germany) and centrifuged.

To study porin response to long-term antibiotic exposure, overnight cultures were diluted and inoculated into LB broth media to obtain a final concentration of 5 × 10^5^ CFU/mL. Bacteria were incubated with sublethal concentrations of antibiotics (kanamycin, tetracycline, chloramphenicol, and carbenicillin) for 16 h at 27 °C and 37 °C. RNAprotect Bacteria Reagent (Qiagen, Hilden, Germany) was added to the culture samples.

Three independent bacterial cultures for each test or control condition were prepared as biological replicates for RNA isolation.

### 3.2. MIC Determination

The MIC for antibiotics against *Y. pseudotuberculosis* strain was determined by the broth microdilution method in a 96-well microplate. Tetracycline (Sigma-Aldrich, St. Louis, MA, USA), carbenicillin (Sigma-Aldrich, St. Louis, MA, USA), kanamycin (Sigma-Aldrich, St. Louis, MA, USA), and chloramphenicol (Sigma-Aldrich, St. Louis, MA, USA) were tested in the concentration range of 0.12–256 μg/mL obtained after a series of 2-fold dilutions in the LB broth. Bacterial colonies were cultured in LB broth at 27 °C to reach McFarland standard 0.5. The suspensions were further diluted and inoculated into the wells with antibiotics to obtain a final concentration of 5 × 10^5^ CFU/mL. The plates were incubated for 16 h at 27 °C and 37 °C. The MIC value was determined as the lowest concentration of an antibiotic at which visible growth was inhibited.

### 3.3. Gene Expression Measurement by qRT-PCR

The expression of *ompF*, *ompC*, *ompA*, *ompX*, *ompY*, *ompR*, *marA47*, and *marA48* genes in *Y. pseudotuberculosis* 488 was studied using qRT-PCR. Total RNA was isolated from the prepared bacterial pellets using the Aurum total RNA mini kit (Bio-Rad, Hercules, CA, USA) according to the manufacturer’s protocol. The RNA concentration and purity were assessed by electrophoresis and a microvolume spectrophotometer (Implen GmbH, Munich, Germany). Of the purified total RNA, 2 μg was reverse-transcribed into cDNA with MMLV RT Kit (Evrogen, Moscow, Russia) and random hexamer primers (Evrogen, Moscow, Russia). The cDNAs were subsequently used to quantify the relative level of *ompF*, *ompC*, *ompA*, *ompX*, *ompY*, *ompR*, *marA47*, and *marA48* by qRT-PCR in Light Cycler 96 (Roche, Basel, Switzerland). The nucleotide sequences of the studied genes are listed in Table S2. The gene-specific primers used in this experiment are listed in Table S3.

RT-PCR was carried out with HS GoTaq Polymerase (Promega, Madison, WI, USA) and the dye Eva Green (Biotium, Fremont, CA, USA). The following thermal cycling parameters were used for the reaction: initial denaturation at 95 °C for 8 min; and 40 cycles of amplification: 95 °C for 15 s, 55 °C for 10 s, and 72 °C for 20 s followed by fluorescence reading. A melting curve was drawn at the end to evaluate the specificity of the PCR. Quantification for each target gene expression was determined by the 2^−ΔΔCT^ method [60] using the 16S rDNA gene as a reference.

qRT-PCR was carried out on three independent biological replicates, each containing two technical replicates. The results are presented in Table S4 as mean ± SD. One-way ANOVA was performed to assess the differences between the means of the test and control groups, with a *p*-value of ≤ 0.05 considered significant.

### 3.4. Porin Gene Sequencing

The coding and regulatory parts of *ompF* and *ompC* genes were amplified using OmpF/C-Bam_for and OmpF/C-Hind_rev primers and sequenced with internal primers (Table S3) to cover all regions. DNA sequencing was done with an ABI 3130 xl automated sequencer (Applied Biosystems, Waltham, MA, USA) and the ABI Prism dye terminator sequencing kit (Applied Biosystems, Waltham, MA, USA) according to the manufacturer’s directions.

### 3.5. GFP Reporter System Construction

The recombinant plasmids pACYC184-F-GFP and pACYC184-C-GFP containing the *Y. pseudotuberculosis ompF* and *ompC* regulatory regions fused to GFP were constructed as previously described [7]. To obtain the coding region of GFP, the plasmid DNA pTurboGFP (Evrogen, Moscow, Russia) was digested with *Bam*HI and *Hind*III (Fermentas, Waltham, MA, USA), and the resulting *gfp* fragment was dephosphorylated with CAP alkaline phosphatase (Fermentas, Waltham, MA, USA). The *ompF*/*ompC* promoter, and terminator regions were amplified using specific primers, which contained *Bam*HI and *Hind*III restriction sites, respectively (Table S3). The resulting PCR products were digested with the same endonucleases and ligated together (separately for *ompF* and *ompC*) with *gfp* in a three-way ligation. Next, ligation mixtures were used as templates for amplifying *ompF*-*gfp* and *ompC*-*gfp* reporter constructions with OmpF-Bam_for/OmpF-Hind_rev or OmpC-Bam_for/OmpC-Hind_rev primers (Table S3). The PCRs were carried out using the following reagents: 2 U of GoTaq DNA polymerase (Promega, Madison, WI, USA), 1× buffer for GoTaq DNA polymerase, 0.2 mM dNTPs, 0.5 μM primers, and 25–50 ng of the template. The following reaction conditions were used: 1 cycle of 5 min at 95 °C for denaturation, 30 cycles of 20 s at 94 °C, 30 s at 55 °C, 1 min 30 s at 72 °C, and 1 final cycle of 5 min at 72 °C. The PCR fragments of expected lengths (1300–1400 bp) were cut from the agarose gel and phosphorylated with T4 polynucleotide kinase (Fermentas, Waltham, MA, USA). The resulting reporter cassettes were inserted in the dephosphorylated vector pACYC184 via *Eco*RV (Figure S1). Recombinant plasmids were transformed into *E. coli* DH5alpha strain by electroporation. The colonies were selected on LB agar plates supplemented with chloramphenicol. All constructs were verified by PCR and DNA sequencing with pACYCSal_seq and pACYCEcV_seq primers (Table S2). The final recombinant plasmids were introduced into competent cells of the *Y. pseudotuberculosis* 488 using electroporation.

### 3.6. Flow Cytometry

To obtain comparable measurements with flow cytometry, the *Y. pseudotuberculosis* 488 containing recombinant plasmid pACYC184-F-GFP or pACYC184-C-GFP were grown in the same conditions as for qRT-PCR analysis. All cultures were incubated in 96-well plates without/with a sublethal concentration of antibiotics (Tet, Km, and Cb) and the addition of chloramphenicol (10 μg/mL) at 37 °C and 27 °C with shaking. After 16 h of cultivation, the cells were washed with PBS, pH 7, and diluted to an OD_600_ of 0.01. The flow cytometry measurements were made with a FACSCanto flow cytometer (BD Biosciences, San Jose, CA, USA). The excitation beam for the GFP was set at 488 nm, and the emission signal was captured with a 530/30 nm bandpass filter. The signals were amplified with the logarithmic mode for SSC, FSC, and fluorescence. For each sample, 100,000 events were recorded. The data were captured with the BD FACSdiva software (version 6.1.2) (BD Biosciences, San Jose, CA, USA), and for data analysis FCS Express 6 (De Novo Software Inc., Los Angeles, CA, USA) was used. The mean fluorescence value of the whole population was chosen as a global indicator of porin expression level in each bacterial culture. *Y. pseudotuberculosis* 488 without plasmids was used as a negative control to exclude background fluorescence.

## 4. Conclusions

In summary, we revealed temporal regulation of *Y. pseudotuberculosis* generalporin genes *ompF* and *ompC* caused by sublethal concentrations of different antibiotics. The porin transcription initially decreased, providing early defensive response of the bacterium, while it returned to that of the untreated cells on prolonged antibiotic exposure. Unlike the major porin genes, the transcription of the alternativeporin genes *ompX* and *lamB* was increased. Such modulation of porin transcription reflects the model of *Y. pseudotuberculosis* physiological adaptation to antibiotic exposure.

The main finding was a phenotypic heterogeneity of *Y. pseudotuberculosis* population manifested in variable porin gene expression under carbenicillin exposure. To verify whether such phenotypic heterogeneity offers a competitive advantage and contributes to low carbenicillin susceptibility for a particular *Y. pseudotuberculosis* subpopulation, future research is warranted.

## Data Availability

Unpublished data are available from the authors.

## Supplementary Materials

The following are available online, Figure S1: GFP reporter constructions; Figure S2: GFP fluorescence intensity in *Y. pseudotuberculosis* 488 transformed with *ompC/ompF* promoter-fused GFP reporter constructs under increasing Cm exposure; Figure S3: GFP fluorescence intensity in *Y. pseudotuberculosis* 488 transformed with *ompF/ompC* promoter-fused GFP reporter constructs at 27 °C and 37 °C incubation temperature; Table S1: MIC of antibiotics against *Y. pseudotuberculosis* 488; Table S2: Nucleotide sequences of *Y. pseudotuberculosis* 488 genes used in the study; Table S3: Oligonucleotide primers used in the study; Table S4: Average Ct values for *ompF*, *ompC*, and 16S rRNA of *Y. pseudotuberculosis* 488.
