# Supplementary material for: Differential Expression of Yersinia pseudotuberculosis General Porin Genes during Short- and Long-Term Antibiotic Stresses
Source: Molecules. 2021 Jun 28;26(13):3956. doi: 10.3390/molecules26133956 (PMC8272246; doi:10.3390/molecules26133956)
Supplement: Supplementary file 1 [file molecules-26-03956-s001.zip › Supplementary_materials.pdf]

Supplementary materials

"Differential expression of *Yersinia pseudotuberculosis* porin genes during short- and long-term antibiotic stresses"

Evgeniya Bystritskaya [1], Nadezhda Chernysheva [1], Anna Stenkova [2], Konstantin Guzev [1], Alexander Rakin [3], and Marina Isaeva [1,2,*]

[1] G.B. Elyakov Pacific Institute of Bioorganic Chemistry, Far Eastern Branch, Russian Academy of Sciences, 159, Pr. 100 let Vladivostoku, Vladivostok 690022, Russia; belyjane@gmail.com (E.B.); chernysheva.nadezhda@gmail.com (N.C.); k.guzev@gmail.com (K.G.)

[2] Far Eastern Federal University, School of Biomedicine, 8 Sukhanova St., Vladivostok 690090, Russia; stenkova@gmail.com (A.S.)

[3] Friedrich-Loeffler-Institut, Federal Research Institute for Animal Health, Institute for Bacterial Infections and Zoonoses, Naumburger Str. 96a, D-07743 Jena, Germany; rakin@gmx.de (A.R.)

* Correspondence: issaeva@gmail.com; Tel.: +7-423-231-11-68

Submitted to Molecules.

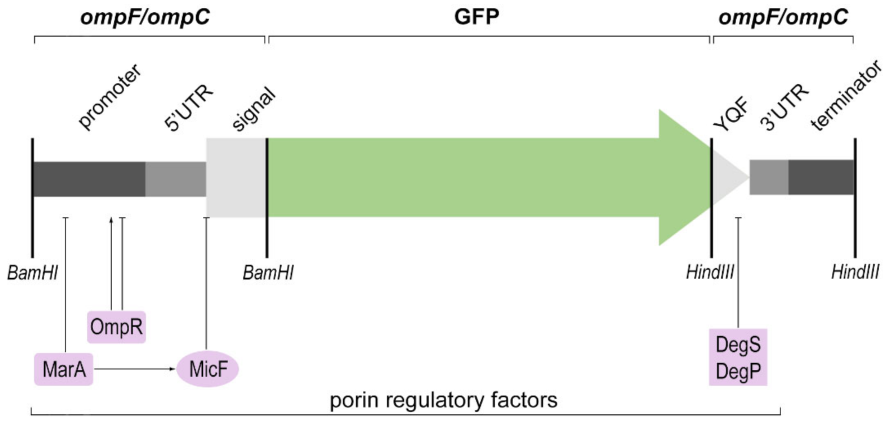

Figure S1. GFP-reporter constructions.

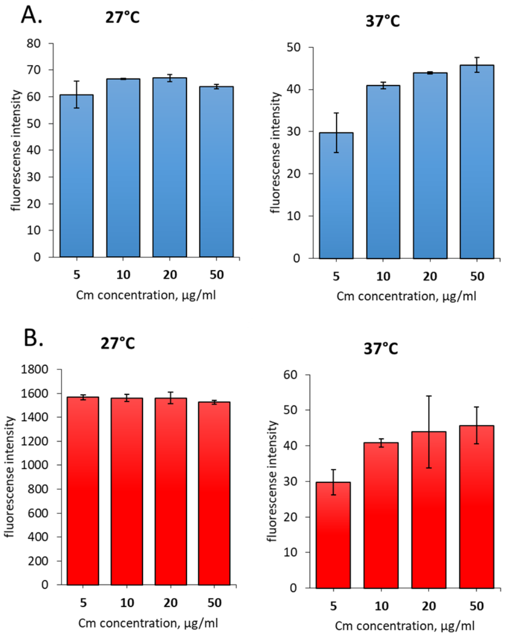

Figure S2. GFP fluorescence intensity in *Y. pseudotuberculosis* 488 transformed with *ompC*/*ompF* promoter-fused GFP reporter constructs under increasing chloramphenicol exposure. (**A**) *ompC*-GFP, (**B**) *ompF*-GFP. All results are expressed as the mean ± standard deviation between three experimental trials.

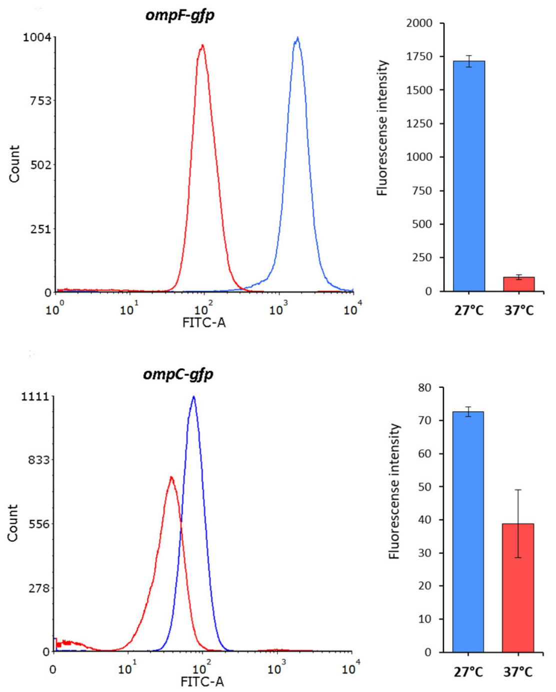

Figure S3. GFP fluorescence intensity in *Y. pseudotuberculosis* 488 transformed with *ompF*/*ompC* promoter-fused GFP reporter constructs at 27 °C and 37 °C incubation temperature. All results are expressed as the mean ± standard deviation between three experimental trials. The histograms from a single representative experiment are shown.

Table S1. MIC of antibiotics against *Y. pseudotuberculosis* 488.

Antibiotic	Class	MIC (μg/ml) at 27 °C	MIC (μg/ml) at 37 °C
kanamycin (Km)	aminoglycosides	4.0	2.0
tetracycline (Tet)	polyketides	1.0	0.5
carbenicillin (Cb)	β-lactams	1.0	1.0
chloramphenicol (Cm)	amphenicols	1.0	1.0

Table S2. Nucleotide sequences of *Y. pseudotuberculosis* 488 genes used in the study (attached in Excel file).

Table S3. Oligonucleotide primers used in the study.

Experiment	Primer name	Sequence	Function
qRT-PCR	OmpF-F	5′-CAAGACGGCAACGCAAC-3′	*ompF* fragment amplification
	OmpF-R	5′-ATGAATCACCACCGAACACT-3′	
	OmpC-F	5′-GACGGGAACCACGACAGT-3′	*ompC* fragment amplification
	OmpC-R	5′-TGACAGGAAGTTATCAGCACC-3′	
	16SRT-F	5′-ATTTAGCCGAGATGCTTTAG-3′	*16S rDNA* frament amplification
	16SRT-R	5′-CTTGATTTCCCACCATTACG-3′	
	MarA47-F	5′-CGCTCTTCCTGTATGGCGAT-3′	*marA47* fragment amplification
	MarA47-R	5′-TAAACGCTCTTCTGCCCCTG-3′	
	MarA48-F	5′-CGGAGAAAGATGACGAGCAAG-3′	*marA48* fragment amplification
	MarA48-R	5′-CAGAGCCAACTGAGGCAGAA-3′	
	OmpR-F	5′-CAAGGTTTTCAGGTCCGCAG-3′	*ompR* fragment amplification
	OmpR-R	5′-ACGGTCCACTTCTTCGCCTT-3′	
	OmpX-F	5′-TGGGTCATGGTCGTTTCACC-3′	*ompX* fragment amplification
	OmpX-R	5′-TTGCGGATACGGCTCTGCTC-3′	
	LamB-F	5′-CCAATAGCACTGGCTGTTGCCG-3′	*lamB* fragment amplification
	LamB-R	5′-TGTTGTTCACCACCGCTTGCCGT-3′	
	OmpA-F	5′-GGGTGGTTTAGTATGGCGTG-3′	*ompA* fragment amplification
	OmpA-R	5′-AACACCAACACTCAGGAGGC-3′	
	OmpY-F	5′-GCCACACTGGGTATCATTC-3′	*ompY* fragment amplification
	OmpY-R	5′-AATAGGTATTTCGCAGCAAC-3′	
fluorescent reporter system construction	OmpF-Bam_for	5′-ttaggatccACGCACGCCGAGAAATGCCA-3′	*ompF* promoter amplification
	OmpF-Bam_rev	5′-ttaggatccAGCTGGGATTACTACTGCAA-3′	
	OmpF-Hind_for	5′-tttaagcttGTCAAACTTAACGAGGCAGTT-3′	*ompF* terminator amplification
	OmpF-Hind_rev	5′-tttaagcttGGCCGATAGACAGAGTAATCT-3′	
	OmpC-Bam_for	5′-ttaggatccGTCAATTTGTGCTAATCATAT-3′	*ompC* promoter amplification
	OmpC-Bam_rev	5′-ttaggatccAATAATGAATGAAAGAACTCGA-3′	
	OmpC-Hind_for	5′-tttaagcttTTCTGATTATAGGCGTGAGT-3′	*ompC* terminator amplification
	OmpC-Hind_rev	5′-tttaagcttGTGAAATCATCACGATGGTGT-3′	
	pACYCSal_seq	5′-ATAAGTGCGGCGACGATAGT-3′	sequencing of reporter inserts
	pACYCEcV_seq	5′-AGGCATAGGCTTGGTTAT-3′	
porin gene sequencing	227-F	5′-GTCTGGGCTTTGCTGGTC-3′	*ompF*
	OmpF-R	5′-ACGGTCCACTTCTTCGCCTT-3′	
	OmpC-2F	5′-ATGGCAGCAGTAGTGAAACC-3′	*ompC*
	OmpC-2R	5′-TAGGCATTGGCACGCTTA-3′	

Table S4. Average Ct values for *ompF*, *ompC* and 16S rRNA of *Y. pseudotuberculosis* 488.

	Condition	16S rRNA average Ct value	*ompF* average Ct value	*ompC* average Ct value
		**short-term antibiotic exposure**		
27 °C	control 1	19.040±0.057	18.840±0.170	17.545±0.177
	control 2	19.240±0.127	19.080±0.240	17.965±0.290
	control 3	19.220±0.156	18.960±0.170	18.080±0.042
	Km treatment 1	18.950±0.141	20.420±0.240	19.285±0.247
	Km treatment 2	18.355±0.205	19.930±0.170	19.220±0.184
	Km treatment 3	18.350±0.057	20.290±0.042	19.440±0.184
	Tet treatment 1	18.670±0.057	19.840±0.141	18.350±0.184
	Tet treatment 2	18.260±0.042	19.995±0.049	18.430±0.113
	Tet treatment 3	17.990±0.085	19.795±0.035	18.375±0.120
	Cb treatment 1	18.550±0.212	19.120±0.212	19.395±0.007
	Cb treatment 2	18.215±0.148	18.915±0.177	18.055±0.205
	Cb treatment 3	17.700±0.100	19.220±0.042	16.805±0.134
	Cm treatment 1	18.535±0.332	18.370±0.297	19.250±0.141
	Cm treatment 2	18.305±0.205	18.520±0.127	18.700±0.085
	Cm treatment 3	18.285±0.092	18.820±0.100	19.190±0.085
37 °C	control 1	17.240±0.099	19.590±0.099	16.665±0.092
	control 2	17.775±0.177	19.715±0.134	16.690±0.170
	control 3	17.290±0.056	19.570±0.085	16.685±0.092
	Km treatment 1	16.580±0.127	20.555±0.290	16.840±0.057
	Km treatment 2	17.100±0.099	21.440±0.226	17.405±0.120
	Km treatment 3	17.375±0.205	21.710±0.297	17.865±0.092
	Tet treatment 1	16.290±0.085	19.585±0.092	18.060±0.113
	Tet treatment 2	16.815±0.049	20.105±0.120	17.675±0.191
	Tet treatment 3	16.775±0.092	20.280±0.127	18.245±0.120
	Cb treatment 1	16.245±0.148	19.090±0.113	16.140±0.198
	Cb treatment 2	15.765±0.148	19.480±0.156	15.180±0.240
	Cb treatment 3	16.805±0.106	21.495±0.120	16.675±0.148
	Cm treatment 1	16.875±0.106	19.260±0.269	18.655±0.106
	Cm treatment 2	16.900±0.014	19.880±0.141	17.880±0.184
	Cm treatment 3	16.995±0.064	20.335±0.205	18.550±0.113
		**long-term antibiotic exposure**		
27 °C	control 1	16.725±0.247	23.590±0.113	22.540±0.057
	control 2	16.965±0.247	24.225±0.007	23.160±0.071
	control 3	16.710±0.240	23.945±0.064	22.805±0.064
	Km treatmen 1t	16.350±0.198	23.275±0.021	23.280±0.042
	Km treatment 2	16.750±0.141	23.410±0.000	23.035±0.078
	Km treatment 3	16.525±0.134	23.375±0.035	23.710±0.042
	Tet treatment 1	17.260±0.170	22.635±0.035	23.310±0.099
	Tet treatment 2	17.380±0.226	23.305±0.134	23.175±0.092
	Tet treatment 3	17.455±0.177	23.590±0.085	23.485±0.092
	Cb treatment 1	17.260±0.085	23.445±0.021	23.165±0.021
	Cb treatment 2	17.465±0.035	24.395±0.007	23.435±0.0919
	Cb treatment 3	17.390±0.113	24.090±0.028	23.330±0.014
	Cm treatment 1	16.990±0.085	22.500±0.000	23.265±0.049
	Cm treatment 2	17.435±0.332	23.250±0.0424	22.920±0.000
	Cm treatment 3	17.195±0.106	23.055±0.049	23.145±0.120
37 °C	control 1	17.245±0.106	25.880±0.014	21.560±0.014
	control 2	17.315±0.049	25.975±0.078	21.490±0.057
	control 3	17.405±0.092	26.020±0.071	21.645±0.049
	Km treatment 1t	17.470±0.000	26.375±0.064	22.285±0.007
	Km treatment 2	17.280±0.170	25.865±0.035	21.915±0.078
	Km treatment 3	17.315±0.092	26.030±0.071	22.085±0.064
	Tet treatment 1	17.700±0.071	26.795±0.120	22.355±0.021
	Tet treatment 2	17.635±0.064	25.865±0.148	22.835±0.035
	Tet treatment 3	17.340±0.099	25.990±0.042	22.375±0.049
	Cb treatment 1	17.675±0.219	26.250±0.099	22.160±0.000
	Cb treatment 2	16.975±0.035	27.145±0.021	22.365±0.021
	Cb treatment 3	17.485±0.106	28.180±0.010	22.975±0.120
	Cm treatment 1	17.205±0.191	25.785±0.318	22.305±0.078
	Cm treatment 2	17.390±0.156	25.415±0.078	21.900±0.057
	Cm treatment 3	17.085±0.106	25.335±0.163	21.980±0.099

Km – kanamycin, Tet – tetracycline, Cb – carbenicillin, Cm – chloramphenicol.
