# Supplementary figures and images for: Differential Expression of Yersinia pseudotuberculosis General Porin Genes during Short- and Long-Term Antibiotic Stresses
Source: Molecules. 2021 Jun 28;26(13):3956. doi: 10.3390/molecules26133956 (PMC8272246; doi:10.3390/molecules26133956)

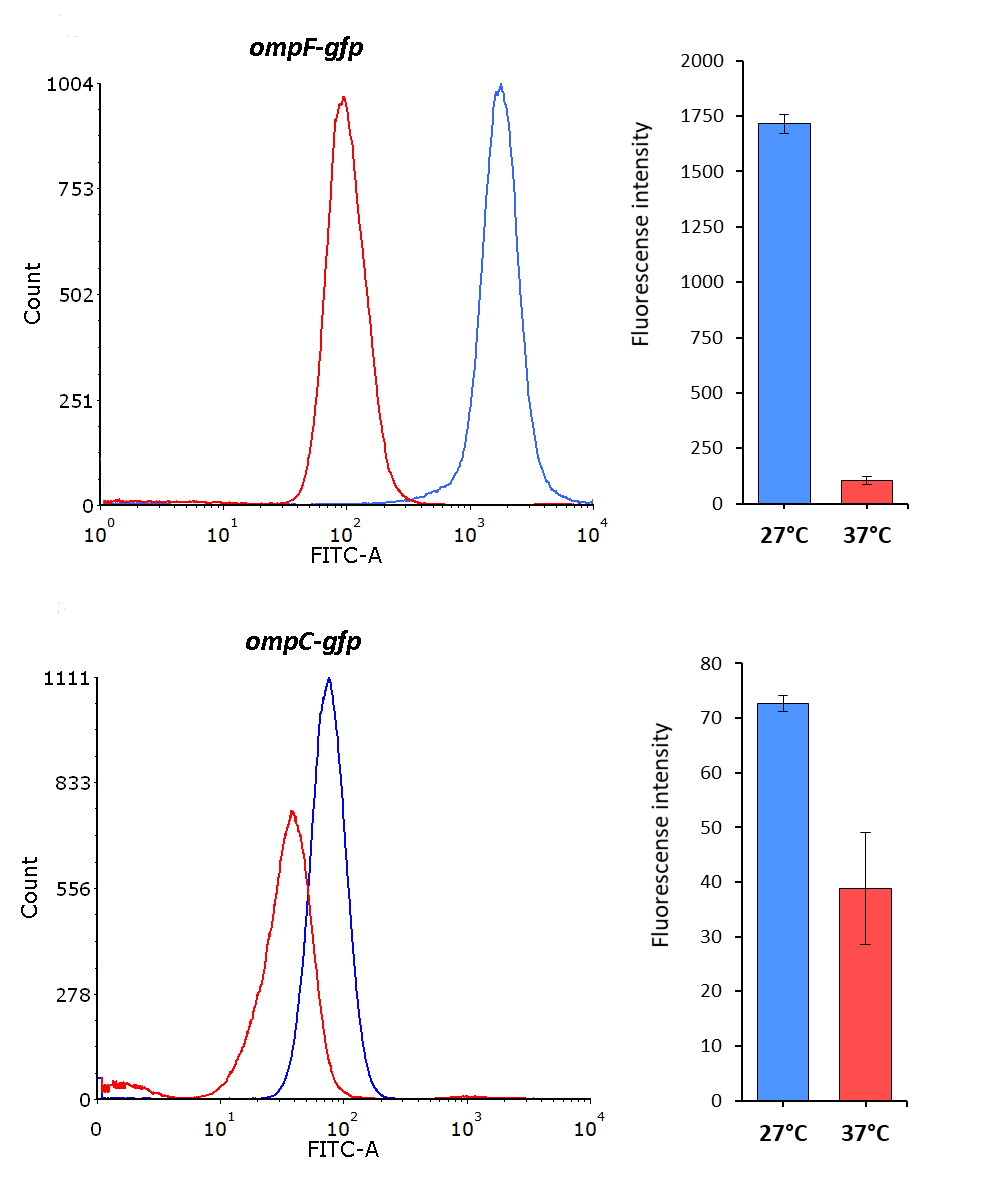

Supplement: Supplementary file 1 [file molecules-26-03956-s001.zip › Figure S3.tiff]

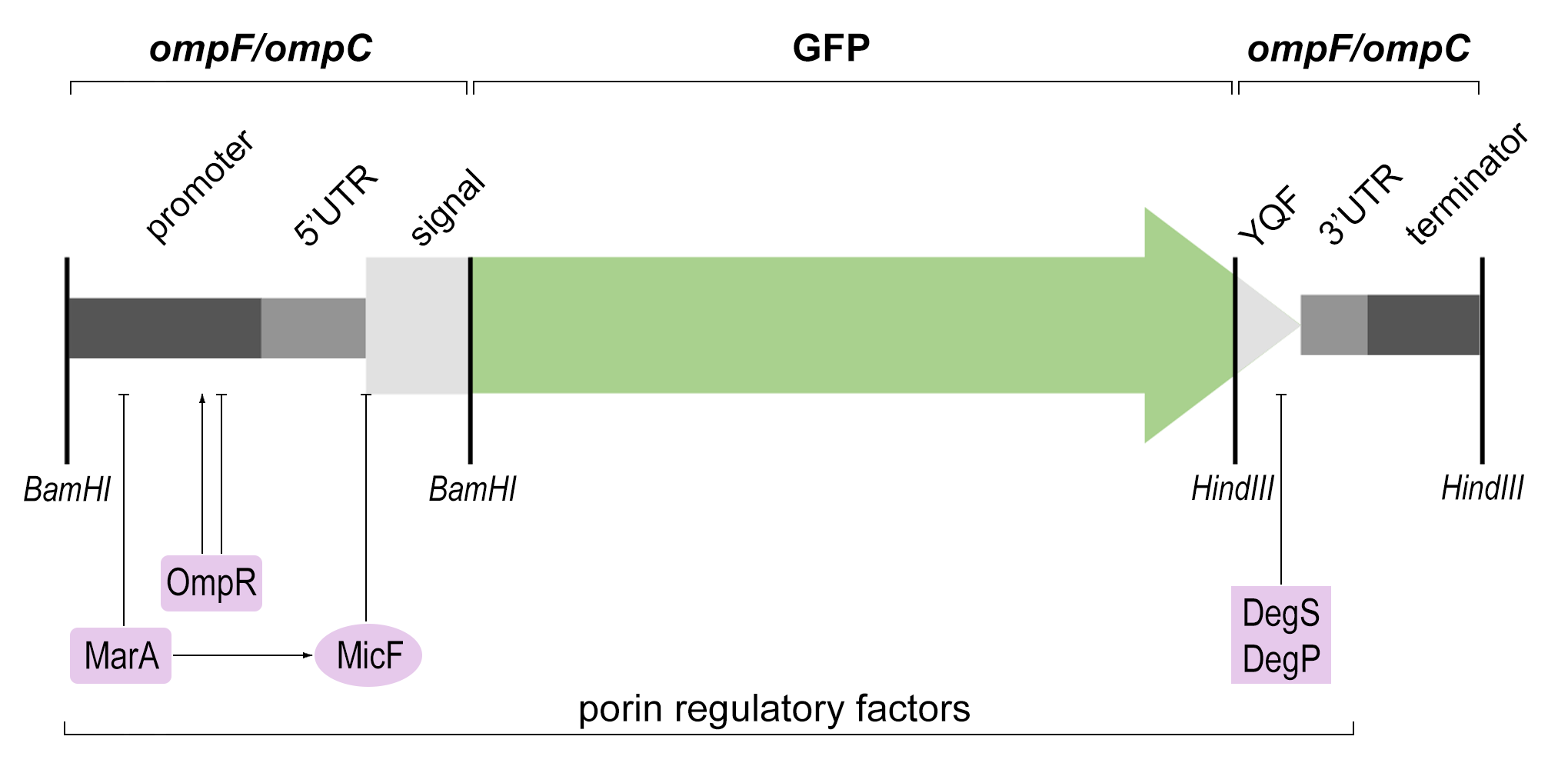

Supplement: Supplementary file 1 [file molecules-26-03956-s001.zip › FigureS1.tif]

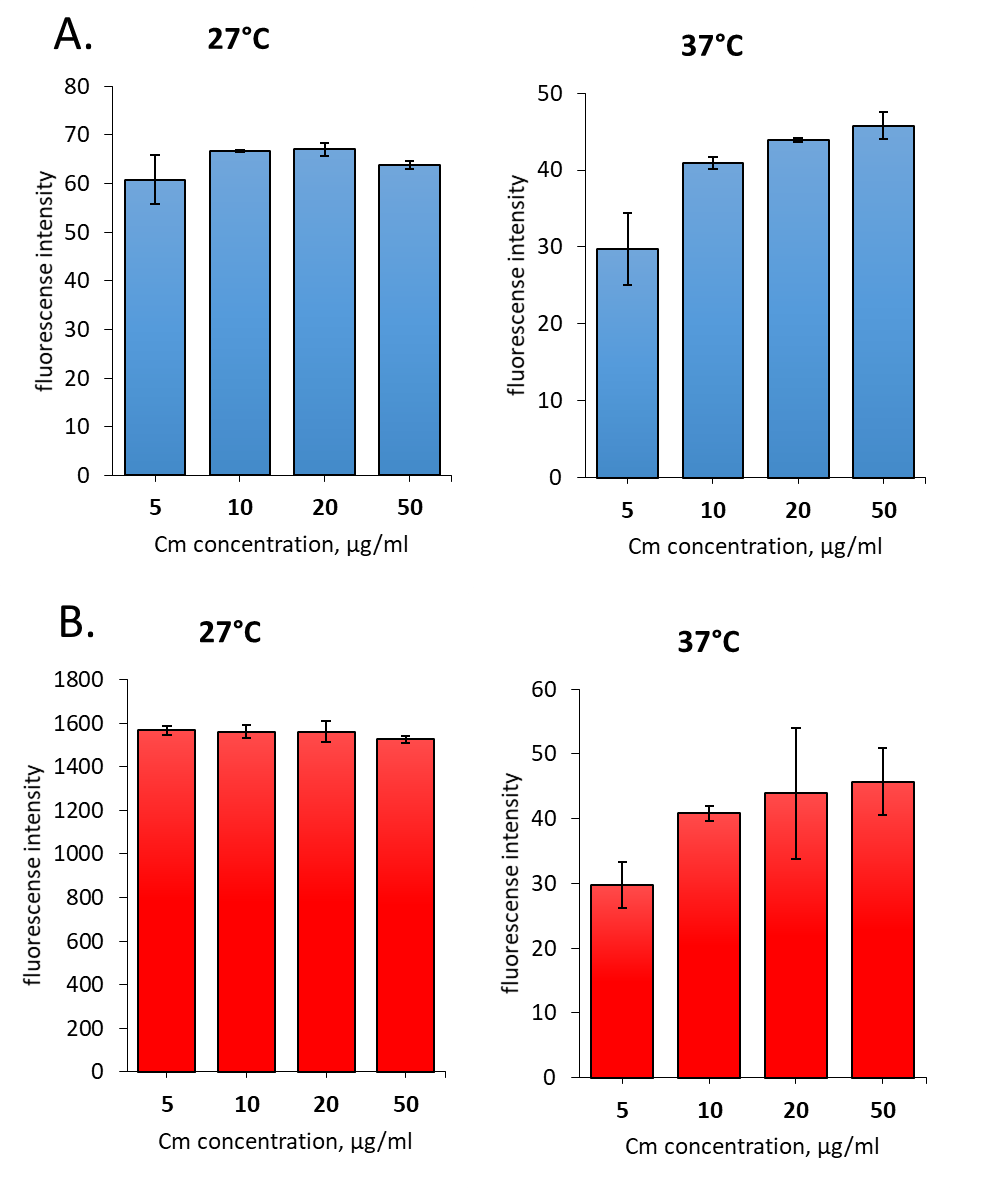

Supplement: Supplementary file 1 [file molecules-26-03956-s001.zip › FigureS2.tiff]
